# Effects of light and the regulatory B-subunit composition of protein phosphatase 2A on the susceptibility of *Arabidopsis thaliana* to aphid (*Myzus persicae*) infestation

**DOI:** 10.3389/fpls.2014.00405

**Published:** 2014-08-21

**Authors:** Brwa Rasool, Barbara Karpinska, Grzegorz Konert, Guido Durian, Konstantin Denessiouk, Saijaliisa Kangasjärvi, Christine H. Foyer

**Affiliations:** ^1^Centre for Plant Sciences, School of Biology, Faculty of Biological Sciences, University of LeedsLeeds, UK; ^2^Molecular Plant Biology, Biocity, University of TurkuTurku, Finland; ^3^Department of Biochemistry, Biocity, University of TurkuTurku, Finland

**Keywords:** aphid fecundity, high light stress, *Myzus persicae*, redox signaling, protein phosphatase, photosynthesis, *Pseudomonas syringae*

## Abstract

The interactions between biotic and abiotic stress signaling pathways are complex and poorly understood but protein kinase/phosphatase cascades are potentially important components. Aphid fecundity and susceptibility to *Pseudomonas syringae* infection were determined in the low light-grown *Arabidopsis thaliana* wild type and in mutant lines defective in either the protein phosphatase (PP)2A regulatory subunit B'γ (*gamma*; *pp2a-b'γ*) or B'ζ (*zeta*; *pp2a-b'ζ1-1* and *pp2a-b'ζ 1-2*) and in *gamma zeta* double mutants (*pp2a-b'γζ*) lacking both subunits. All the mutants except for *pp2a-b'ζ 1-1* had significantly lower leaf areas than the wild type. Susceptibility to *P. syringae* was similar in all genotypes. In contrast, aphid fecundity was significantly decreased in the *pp2a-b'γ* mutant relative to the wild type but not in the *pp2a-b'γζ* double mutant. A high light pre-treatment, which led to a significant increase in rosette growth in all mutant lines but not in the wild type, led to a significant decrease in aphid fecundity in all genotypes. The high light pre-treatment abolished the differences in aphid resistance observed in the *pp2a-b'γ* mutant relative to the wild type. The light and CO_2_ response curves for photosynthesis were changed in response to the high light pre-treatment, but the high light effects were similar in all genotypes. These data demonstrate that a pre-exposure to high light and the composition of B-subunits on the trimeric PP2A holoenzymes are important in regulating plant resistance to aphids. The functional specificity for the individual regulatory B-subunits may therefore limit aphid colonization, depending on the prevailing abiotic stress environment.

## Introduction

Plants respond to environmental stress through a complex signaling network involving stress receptors and hormones, plant growth regulators, calcium, and protein kinase cascades (Bostock, 2005; Pieterse et al., 2009; Verhage et al., 2010; Atkinson and Urwin, 2012). Identifying genes, processes, and regulators associated with plant stress responses will not only allow a deeper understanding of plant stress tolerance but also provide new opportunities for developing broad-spectrum stress tolerant crop plants. Cross-tolerance to environmental stresses is a common phenomenon that allows resistance to a range of different stresses upon exposure to only one type of stress (Pastori and Foyer, 2002; Mittler, 2006). Cross-tolerance occurs because of synergistic co-activation of non-specific stress-responsive pathways that cross biotic-abiotic stress boundaries (Bostock, 2005). In many cases, cross-tolerance has been linked to enhanced production of reactive oxygen species (ROS) and oxidative signaling (Foyer and Noctor, 2009). ROS-production and processing systems are intrinsically-linked to the plant response to infestation by insects, including phloem feeders such as aphids (Kerchev et al., 2012).

Aphids penetrate plant tissues to feed on photo-assimilates in the phloem by probing between the cells in the epidermal and mesophyll layers with their piercing-sucking mouthparts called stylets (Nam and Hardie, 2012). In addition to causing damage to tissues, aphid feeding transmits disease-causing viruses and fungi (Pimental, 2004). For example, the green peach aphid (*Myzus persicae*) is classed as “generalist” feeder because it can colonize more than 30 plant families. Moreover, *M. persicae* transmits over 100 viruses (Van Emden et al., 1969). A recent investigation of the responses of *A. thaliana* to *Myzus persicae* revealed that aphid attack resulted in rapid changes to the leaf transcriptome and metabolome signature, presenting evidence for the involvement of redox, salicylic acid (SA), and abscisic acid signaling pathways (Kerchev et al., 2013). The pattern recognition receptors in plants to induce the first stages of immunity to pathogens (sometimes called pathogen-associated molecular pattern-triggered immunity) are either RLKs or receptor-like proteins. BRASSINOSTEROID INSENSITIVE1-ASSOCIATED KINASE1/SOMATIC-EMBRYOGENESIS RECEPTOR-LIKE KINASE3 (BAK1/SERK3), is a plasma membrane leucine-rich repeat receptor like kinase that is required for ROS induction and callose deposition by *M. persicae* elicitors (Prince et al., 2014). Such studies suggest that innate immunity to aphids, as with resistance to other stresses, involves early perception of elicitors by cell surface-localized pattern recognition receptors leading to subsequent downstream immune signaling that involves redox signaling and protein kinase/phosphatase cascades (Kangasjärvi et al., 2012; Prince et al., 2014). The following studies were performed to analyse the role of trimeric protein phosphatase 2A (PP2A) in the plant response to aphid infestation because a cytoplasmic regulatory PP2A B'γ subunit was recently identified as a key component controlling pathogenesis responses in *A. thaliana* (Trotta et al., 2011; Li et al., 2014). Knockdown of the specific B'γ subunit resulted in a lesion mimic phenotype with constitutive activation of jasmonic acid and SA related defense pathways, premature disintegration of chloroplasts and cell death upon aging (Trotta et al., 2011). The constitutive immune responses of *pp2a-b'γ* were highly conditional, and became observable when the plants were grown in 50% relative humidity and moderate light intensity, but not when grown under high light (Trotta et al., 2011; Li et al., 2014). Proteomic studies suggested that the PP2A-B'γ dependent signaling effects involve antioxidant enzymes, such as copper/zinc superoxide dismutase 2 (CSD2) and monodehydroascorbate reductase 2 (MDAR2), with well-known roles in the maintenance of cellular ROS homeostasis (Trotta et al., 2011; Li et al., 2014). These observations also suggest that PP2A-B'γ is a focal point of cross-talk between plant responses to pathogens and light (Trotta et al., 2011; Li et al., 2014). In the following studies therefore we analyzed the role of PP2A in the responses to aphid infestation of *Arabidopsis thaliana* plants grown under either low or high light.

Trimeric PP2A phosphatases are composed of a catalytic subunit C, a scaffold subunit A, and a highly variable regulatory subunit B. In the *A. thaliana* genome these subunits are encoded by 5, 3, and 17 distinct genes, respectively, (Farkas et al., 2007; Sents et al., 2013). The catalytic C subunit of PP2A attains an active conformation only upon dimerization with a scaffold subunit A. Formation of a PP2A-A/C dimer in turn forms a platform for interaction with the regulatory B subunit, which is thought to determine the sub-cellular localization and target specificity of the PP2A holoenzyme (Farkas et al., 2007; Matre et al., 2009; Uhrig et al., 2013). It has also been proposed that the large number of isoforms for each subunit could provide extensive variability in subunit combinations, allowing versatile but highly specific functions for PP2A in the dephosphorylation of specific target proteins. Computational modeling predicted that both PP2A-B'γ and PP2A-B'ζ could interact with any of the PP2A A and C subunits, and therefore hold the potential to form similar PP2A trimers. However, the functional interactions between regulatory B-subunits remain poorly understood. We therefore examined the structural properties of PP2A in relation to the ability of *A. thaliana* to resist infestation by *M. persicae* in plants that had been grown under low light or had been given a pre-exposure to high light. The data presented here demonstrate that the B'γ subunit composition is an important determinant of the ability of the plants to limit aphid infestation at low but not at high light. Moreover, we show exposure to high light leads to cross tolerance responses that limit aphid fecundity in all genotypes.

## Materials and methods

### Plant material

Homozygote *pp2a-b'γ* (SALK_039172 for At4g15415), *pp2a-b'ζ1-1*, and *pp2a-b'ζ 1-2* (SALK_107944C and SALK_150586 for At3g21650, respectively) mutant lines were identified from the SALK institute's collection by PCR analysis according to the institute's protocols (Alonso et al., 2003). A *pp2a-b'γζ* double mutant was constructed by crossing the SALK_039172 and SALK_107944C single mutants and selecting homozygotes from the F2 generation using the same set of PCR primers that were used to screen for the single *pp2a-b'γ* and *pp2a-b'ζ1-1* mutants. Insertion mutant information was obtained from the SIGnAL website at http://signal.salk.edu. To assess the expression of *PP2A-B'ζ* and *PP2A-B'γ* in the mutant lines, RNA was isolated with Agilent Plant RNA Isolation Mini Kit (product number 5185-5998) and thereafter DNAse-treated with Ambion DNA-*free* Kit (product number AM1906) according to the manufacturer's instructions for rigorous DNase treatment. One microgram of RNA was used for cDNA synthesis using the Invitrogen SuperScript III First-Strand Synthesis Super Mix for qRT-PCR (product number 11752-050). cDNAs of specific genes were amplified using Thermo Scientific Phire Hot Start II DNA Polymerase (product number F-122S). To confirm the absence of *PP2A-B'ζ* mRNA in *pp2a-b'ζ1-1, pp2a-b'ζ1-2*, and *pp2a-b'γζ* double mutant, the cDNA was used for RT-PCR with the primers FOR (5′-TGCCTATAGTCTTCCCAGCTCT-3′) and REV (5′GTGGACTCAGAGCTGCTTGT-3′). *pp2a-b'γ* is a knock-down mutant with 40% reduction in *PP2A-B'γ* transcript level (Trotta et al., 2011). To assess the abundance of *PP2A-B'γ* mRNA in *pp2a-b'γ, pp2a-b'ζ1-1*, and *pp2a-b'γζ* double mutant, PP2A-B'γ cDNA was specifically amplified with the primers FOR (5′-TGTGTTGCGTTGTGTTCGAC-3′) and REV (5′-GGTGCACCATGAATTTCCCG-3′) and normalized to *Actin 2* amplified with the primers FOR (5′-GTGAACGATTCCTGGACCTGCCTC-3′) and REV (5′-GAGAGGTTACATGTTCACCACAAC-3′). DNA bands stained with NIPPON Genetics Midori Green Advanced DNA stain (product number MG 04) in 1% agarose gels were detected with the PerkinElmer Geliance 1000 Imaging System. Band intensities were analyzed with ImageJ.

Unless otherwise stated *Arabidopsis thaliana* ecotype Columbia wild type and the mutant genotypes were grown in compost (SHL professional potting compost) in controlled environment chambers under an 8 h/16 h day/night regime, with an irradiance of 250 μmol m^−2^ s^−1^ (low light conditions). The relative humidity was 60% and day/night temperatures were 20°C.

### Amino acid alignment and construction of phylogenetic tree

Amino acid alignment was conducted using ClustalX program with gap penalty 10 and gap extension penalty set to 0.2. The amino acid sequence distances were inferred by using the Maximum Likelihood method based on the JTT matrix-based model (Jones et al., 1992). The tree with the highest log likelihood (−8780.5476) is shown. Initial tree(s) for the heuristic search were obtained automatically as follows. When the number of common sites was <100 or less than one fourth of the total number of sites, the maximum parsimony method was used; otherwise BIONJ method with MCL distance matrix was used. The tree was drawn to scale, with branch lengths measured in the number of substitutions per site. The analysis involved 17 amino acid sequences from Arabidopsis and the human ortholog B56γ. All positions containing gaps and missing data were eliminated. There were a total of 381 positions in the final dataset. Evolutionary analyses were conducted in MEGA5 (Tamura et al., 2011).

### Structural modeling

Known X-ray structures of individual proteins were obtained from the Protein Data Bank (http://www.rcsb.org; Berman et al., 2000). Protein folds were assigned according to the SCOP database (http://scop.mrc-lmb.cam.ac.uk/scop; Murzin et al., 1995). Programs for protein structure modeling, Modeler (Sali and Blundell, 1993) and Homodge (in Bodil; Lehtonen et al., 2004) were used for homology modeling of domains based on known related structures. The Basic Local Alignment Search Tool (BLAST) at the National Center for Biotechnology Information was used to search regions of sequence homology (http://www.ncbi.nlm.nih.gov/blast/). Discovery Studio (http://accelrys.com) and Sybyl (http://www.tripos.com) molecular modeling environments were used for additional modeling, structure superpositions, structure viewing and analysis. Identification of local atom environments, polar and non-polar interactions and contacts between amino acids were made using the CSU Analysis of Interatomic Contacts in Protein Entries software package (http://bip.weizmann.ac.il/oca-bin/lpccsu/), and the Automated Analysis of Interatomic Contacts software (Sobolev et al., 1999).

**Figure 1:**
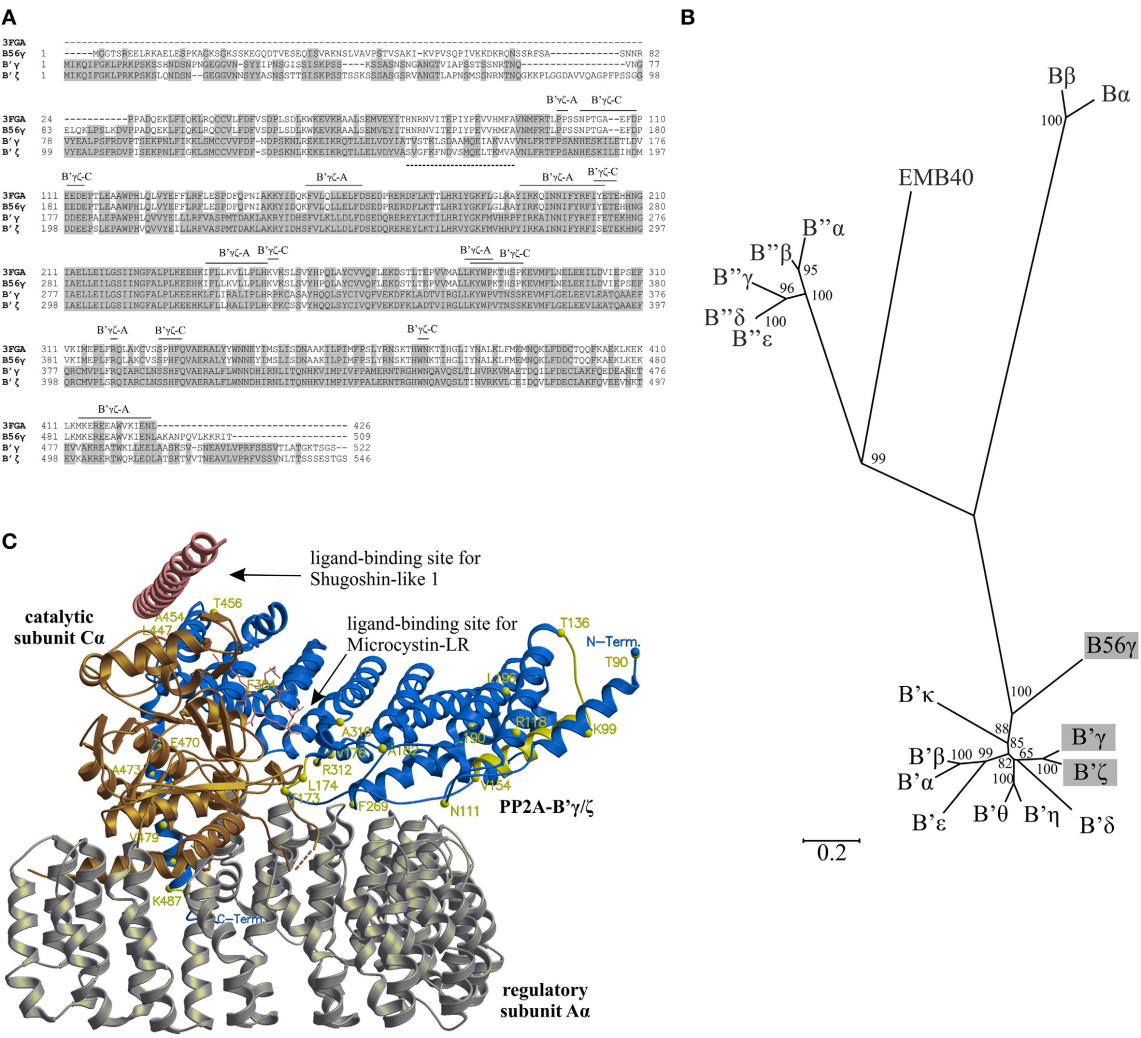
**Structural properties of PP2A-B'γ and PP2A-B'ζ. (A)** Amino acid sequence alignment between *A. thaliana* PP2A-B'γ (B'γ) and PP2A-B'ζ (B'ζ), human PP2A-B56γ, and the sequence of PP2A-B56γ from the X-Ray structure (PDB Code: 3FGA). Gray areas indicate sequence identity and the lines indicate areas of interactions between PP2A-B'γ or PP2A-B'ζ and the PP2A-A and PP2A-C subunits, based on the structure of 3FGA. The dashed line indicates a variable segment between PP2A-B'γ and PP2A-B'ζ. Bα, AT1G51690; Bβ, AT1G17720; B'α, AT5G03470; B'β, AT3G09880; B'γ, AT4G15415; B'δ, AT3G26030; B'ε, AT3G54930; B'ζ, AT3G21650; B'η, AT3G26020; B'θ, AT1G13460; B'κ, AT5G25510; B”α, AT5G44090; B”β, AT1G03960; B”γ, AT1G54450; B”δ, AT5G28900; B”ε, AT5G28850; EMB40, AT5G18580; B56γ, Q13362. **(B)** Unrooted phylogeny tree showing evolutionary relationship between Arabidopsis PP2A regulatory B subunits and their human ortholog B56γ. The bar corresponds to substitutions per amino acid. The positions of Arabidopsis PP2A-B'γ and PP2A-B'ζ and human B56γ are highlighted with gray. **(C)** Structural model of a trimeric PP2A complex containing Arabidopsis PP2A-B'γ (blue), mouse PP2A-Aα (silver), and human PP2A-Cα (gold). Molecular modeling was based on the sequence alignment shown in **(A)**, using the structure 3FGA as a template. Sites of amino acid differences between PP2A-B'γ and PP2A-B'ζ are mapped in yellow. The N-terminal segment M1-V88 of PP2A-B'γ is not present in the structural model. All amino acid labels are as in PP2A-B'γ. Sites known to recognize the Shugoshin-like 1 and Microcystin-LR molecules in 3FGA are indicated.

The PP2A holoenzyme (PDB code: 3FGA) composed of regulatory subunit α (Aα; *M. musculus*)/regulatory subunit γ (B56γ; *H. sapiens*)/catalytic subunit α (Cα; *H. sapiens*) bound to the synthetic construct microcystin LR *(Microcystis aeruginosa)* and to the 47 amino acid fragment of Shugoshin-like 1 protein (Sgo1, *H. sapiens*), at 2.7 Å was chosen as a template to model the Arabidopsis PP2A triple complex. Other available PP2A structures include (1) the PP2A holoenzyme (Aα/B56γ/Cα; *H. sapiens*) bound to the synthetic construct microcystin LR from Cyanobacteria at 3.3 Å (PDB codes: 2NPP, 2NYM, and 2NYL) and (2) the PP2A holoenzyme (Aα; *M. musculus*)/(B56γ; *H. sapiens*)/(Cα; *H. sapiens*) bound to the synthetic construct microcystin LR from *M. aeruginosa* at 3.5 Å (PDB codes: 2IAE). Amino acid sequences of Arabidopsis AT4G15415 and AT3G21650 (NCBI protein codes: NP_567464.1 and NP_188802.1) were taken to model the PP2A-B'γ and PP2A-B'ζ subunits, respectively. The AT1G10430 (NCBI protein code: NP_172514.1) sequence was taken to model PP2A-C2, and the AT1G25490 (NCBI protein code: NP_173920.1) sequence was taken to model PP2A-A1. The alignments used for modeling of PP2A-B'γ/B'ζ, PP2A-A, and PP2A-C are shown in Figure 1A, Supplemental Figures 1, 2, respectively.

### High light treatments

Wild type and mutant plants were grown as described above under low light conditions (250 μmol m^−2^ s^−1^) for 2 weeks in controlled environment chambers then half of the plants weregrown for a further 7 days under low light conditions in the controlled environment chambers and half were transferred to duplicate controlled environment cabinets in which the light intensity was 800 μmol m^−2^ s^−1^(high light) and grown for a further 7 days. In both treatments, the day and night temperatures in the controlled environment chambers were carefully monitored and maintained at 20°C, with a relative humidity was 60%. In all cases, photosynthesis and aphid fecundity measurements were performed between 3 and 6 h into the photoperiod.

### Shoot growth determination

Leaf area was measured on 3 week-old plants. Photographs were taken with a Canon EOS 450 D (Canon Inc., Tokyo, Japan). Leaf area was measured and analyzed using Fiji ImageJ (http://fiji.sc/).

### Photosynthetic gas exchange measurements

Photosynthetic gas exchange measurements were performed on whole rosettes that had been grown for 2 weeks under low light conditions (250 μmol m^−2^ s^−1^) and then grown for a further 7 days either under low light or under high light (800 μmol m^−2^ s^−1^) conditions. Photosynthetic CO_2_ assimilation rates and intracellular CO_2_ (Ci) concentrations were measured using a portable Photosynthetic System (LI-6400XT) LI-COR at 20°C in the leaf chamber with a light intensity of (250 μmol m^−2^ s^−1^) and an atmospheric CO_2_ concentration of 400 μmol mol^−1^. In all cases, rosettes were allowed to acclimatize to the chamber for 15 min prior to measurement to allow stabilization of parameters. Measurements were made on 5 plants per line per experiment.

### Chlorophyll *A* fluorescence quenching parameters

The ratio of dark adapted variable chlorophyll *a* fluorescence (Fv) to the maximal value of chlorophyll a fluorescence (Fm) in the dark adapted state was measured in the leaves of 3 week-old plants following the transfer from LL growth conditions to HL conditions using a FluorPen, which is a portable, battery-powered fluorometer (FP 100-SN-FP-680 Equipements Scientifiques S.A., Nanterre, France).

### Photosynthetic pigments

Leaves were harvested at the points indicated on the figures and weighed. Samples (100 mg fresh weight) were ground in liquid nitrogen. Photosynthetic pigments were extracted in 95% ethanol and determined according to the method of Lichtenthaler (1986).

### Aphid culture conditions

Green peach aphids (*Myzus persicae* Sulzer) derived from stocks that had been collected in Scotland in the years 2002–2004 were obtained from Dr. Robert Hancock, James Hutton Institute, Invergowrie, UK. Aphid stocks were maintained on mature potato plants in transparent cages in an insectary under controlled environment conditions (16 h photoperiod and day/night temperatures of 20°C).

### Aphid fecundity

Aphid fecundity was determined on 3 week-old plants by the method of Fenton et al. (2010). A single 1-day-old nymph was placed in the center of a leaf (per plant) and was enclosed in a mesh perspex® cage (5 cm internal diameter) capped with a thin mesh (mesh size 200 μm). Plants with cages were then returned to the low light controlled environment chamber. After 15 days the total number of aphids was counted. Each fecundity experiment involved 15 plants per genotype per experiment and was repeated 3 times.

### *Pseudomonas* inoculation

For these experiments, the growth conditions for the wild type and the mutant genotypes were as described above except that the growth irradiance was 130 μmol m^−2^ s^−1^. The virulent *Pseudomonas syringae* pv tomato strain DC3000 (Pst) was grown overnight in NYGA medium with 10 mg/mL tetracycline and 100 mg/mL rifampicin as in Trotta et al. (2011). Bacterial suspensions were washed twice in 10 mM MgCl_2_, diluted to 10^5^ colony forming units ml^−1^ and carefully infiltrated into fully expanded leaves using a needleless syringe on the abaxial surface. Four days post-infection, bacterial growth cm^−2^ was determined in leaf material homogenized in 10 mM MgCl_2_ to liberate the bacteria. Serial dilutions of the homogenates were plated (in duplicate) on NYGA medium supplemented with 10 mg/mL tetracycline and 100 mg/mL rifampicin. Colonies on the plates were counted after incubation at 28°C for 24 h.

### Statistical analysis

Data represent the mean ± standard error of the mean (SEM). Statistical analysis was performed by Student's *t*-test and a One-Way ANOVA (IBM SPSS Statistics—version 20). The values were considered statistically different when *P* was < 0.05.

## Results

### PP2A-B'γ and PP2A-B'ζ may form similar PP2A trimers

Pairwise alignment of amino acid sequences revealed that Arabidopsis PP2A-B'γ and PP2A-B'ζ share 80% sequence identity (Figure 1A), and phylogenetic clustering of PP2A-B subunits illustrated a close evolutionary relationship of PP2A-B'γ and PP2A-B'ζ with their human (*Homo sapiens*) counterpart B56γ (Figure 1B, Supplemental Figure 1). The areas with greatest sequence variation between PP2A-B'γ and PP2A-B'ζ reside within the N and C terminal ends, with a unique 18 amino acid insertion present only in PP2A-B'ζ (Figure 1A). Additionally, the two proteins differ within a short segment between T136-V154 and S157-A175 in PP2A-B'γ and PP2A-B'ζ, respectively, (Figure 1A). In the middle parts of the proteins, only localized single amino acid differences occur between PP2A-B'γ and PP2A-B'ζ (Figure 1A), and these highly conserved areas incorporate all predicted sites of interaction with PP2A-A and PP2A-C, suggesting that PP2A-B'γ and PP2A-B'ζ may form similar PP2A trimers.

Next we analyzed structural differences and similarities between PP2A-B'γ and PP2A-B'ζ within putative PP2A complexes. The A and C subunits that associate with B'γ or B'ζ in PP2A heterotrimers have not yet been identified. Nevertheless, since PP2A-A and PP2A-C subunits are evolutionary highly conserved (Supplemental Figures 2, 3), their mammalian counterparts present in a chimeric mouse (*Mus musculus*)/human PP2A (PDB code: 3FGA) could serve as a template for molecular modeling (Figure 1C). With these structural models, we aimed to dissect (1) whether the inter-subunit interacting areas between PP2A-B'γ and PP2A-B'ζ differ from each other and (2) whether these interacting areas would specifically interact with distinct *A. thaliana* PP2A-A and PP2A-C subunits only.

The very N-terminal regions of PP2A-B'γ and PP2A-B'ζ could not be modeled, since they were not present in any of the PP2A X-Ray structures available. However, it is likely that the N-terminal segment M1-V88 resides outside of the inter-subunit interface of PP2A, and interacts with the variable segment T136-V154, which forms an α-helix flanking the N-terminal end of PP2A-B'γ and PP2A-B'ζ (Figure 1C). The N-terminal regions in either PP2A-B'γ and PP2A-B'ζ are therefore likely to form separate domains, which do not contribute to the inter-subunit communication within PP2A trimers.

Most single amino acid substitutions scattered across the PP2A-B'γ and PP2A-B'ζ sequences are either equivalent without major differences in chemical properties or reside outside of the PP2A inter-subunit interfaces (Figures 1A,C). Thus, the structural properties of the inter-domain interacting areas in PP2A-B'γ and PP2A-B'ζ would allow formation of similar PP2A trimers. The corresponding interacting areas of PP2A-A and PP2A-C lie within their regions of high identity, and are therefore unlikely to affect the formation of trimeric PP2A holoenzymes. Additionally, the variable N-terminal domains of PP2A-B'γ and PP2A-B'ζ may target PP2A holoenzymes to specific target proteins, thereby allowing functional specificity for the individual regulatory B-subunits.

### Effects of light and PP2A subunit composition on shoot growth

**Figure 2:**
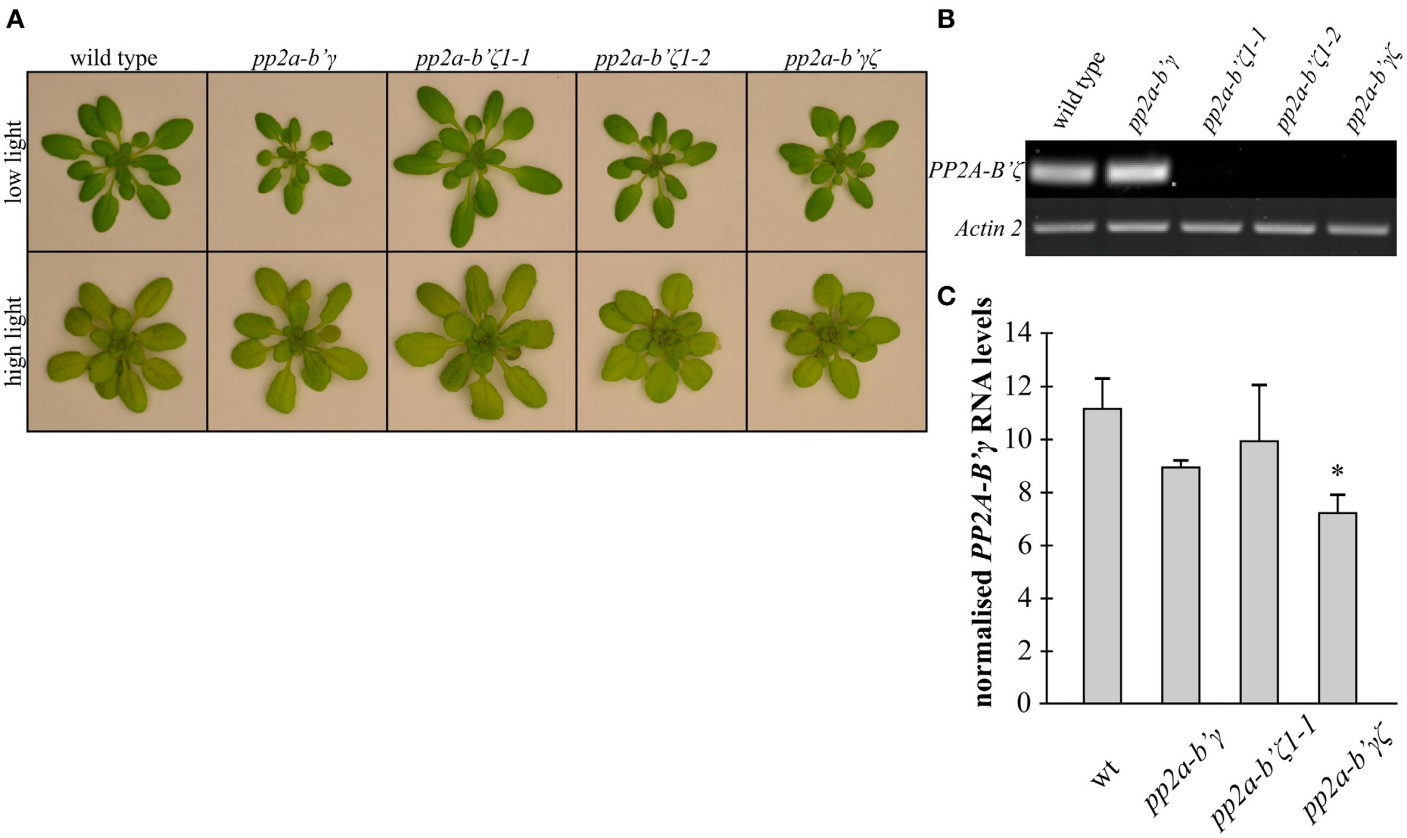
**A comparison of the rosette phenotypes of the *pp2a-b'γ, pp2a-b'ζ1-1, pp2a-b'ζ 1-2*, and the *pp2a-b'γζ* double mutant grown under low and high light conditions. (A)** Plants grown for 2 weeks under low light (250 μmol m^−2^ s^−1^) and then either maintained for a further 7 days under low light growth conditions (top row) or transferred to high light (800 μmol m^−2^ s^−1^) for 7 days (bottom row). A *pp2a-b'γζ* double mutant was constructed by crossing the SALK_039172 (*pp2a-b'γ*) and SALK_107944C (*pp2a-b'ζ 1-1*) single mutants. **(B)** RT-PCR analysis of *PP2A-B'ζ* mRNA in wild type and *pp2a-b'γ, pp2a-b'ζ1-1, pp2a-b'ζ1-2*, and the *pp2a-b'γ* ζ double mutant. Amplification of *Actin 2* is used to control for equal cDNA-levels. **(C)** RT-PCR analysis of *PP2A-B'γ* mRNA in wild type, *pp2a-b'γ, pp2a-b'ζ1-1*, and the *pp2a-b'γ* ζ double mutant. Band intensity values of the *PP2AB'γ*-amplicon of each sample were normalized to *Actin 2 values*. Average values ± standard error values are shown (*n* = 3–4). The asterisk denotes a value that is significantly lower than the wild type-value (*P* < 0.05) as determined by a Student's *t*-test.

When wild type and mutant plants were grown for 3 weeks under low light (250 μmol m^−2^ s^−1^) the rosettes of all of the mutant genotypes except for *pp2a-b'ζ1-1* were visibly smaller than the wild type at 3 weeks (Figure 2A). However, when the plants were grown for 2 weeks under low light (250 μmol m^−2^ s^−1^) and then transferred for 7 days to high light (800 μmol m^−2^ s^−1^), the rosette phenotypes were more similar in all genotypes than under low light, although the *pp2a-b'ζ1-2* mutant and the *pp2a-b'γζ* double mutant were visibly smaller than the other lines under high light conditions (Figure 2A). Knockout of PP2A-B'ζ resulted in a strong depletion of PP2A-B'ζ transcripts in pp2a-b'ζ 1-1, pp2a-b'ζ 1-2, and the pp2a-b'γζ double mutants (Figure 2B). Knockdown *pp2a-b'γ* in turn harbors a T-DNA insertion in its promoter region (Trotta et al., 2011), and resulted in a 20% decline in the level of PP2A-B'γ mRNA in knockdown *pp2a-b'γ* and 35% in *pp2a-b'γζ* double mutants as compared to wild type plants (Figure 2C).

**Figure 3:**
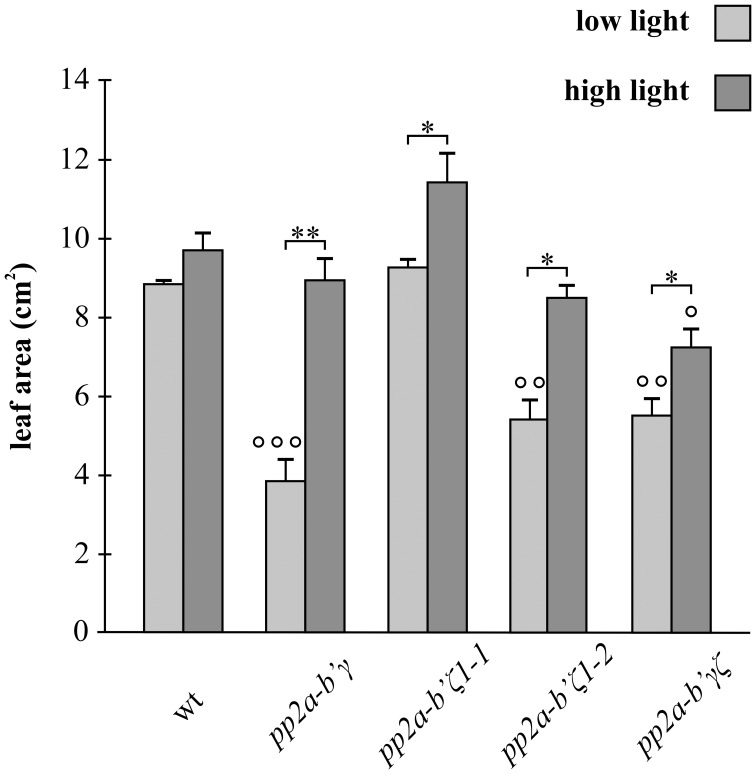
**A comparison of the rosette leaf areas of the *pp2a-b'γ, pp2a-b'ζ 1-1, pp2a-b'ζ 1-2*, and the pp2a-*b'*γζ double mutant grown under low and high light conditions**. Leaf area measurements were performed on plants that had been grown for 2 weeks under low light (250 μmol m^−2^ s^−1^) and then either maintained for a further 7 days under low light growth conditions or transferred to high light (800 μmol m^−2^ s^−1^) for 7 days. The results are average values ± standard error values, *n* = 5. ^*^p < 0.05; ^**^p < 0.01 in Significance given from analysis by student's *t*-test and One-Way ANOVA analysis of LL and HL values for each genotype, as follows ° *p* < 0.05; °° *p* < 0.01; °°° *p* < 0.001 in Student's *t*-test and One-Way ANOVA comparisons between the mutant lines and wild type under LL or HL light conditions.

Leaf area measurements were performed on plants that had either been grown for 3 weeks under low light (250 μmol m^−2^ s^−1^) or for 2 weeks under low light followed by 7 days under high light (800 μmol m^−2^ s^−1^; Figure 3). The wild type plants had similar leaf areas under both high and low light growth conditions (Figure 3). In contrast, the leaf area was significantly increased in all of the mutant genotypes under high light relative to low light conditions (Figure 3). The high light-dependent increase in leaf area was most marked in the *gamma* (*pp2a-b'γ*) mutants (Figure 3).

**Figure 4:**
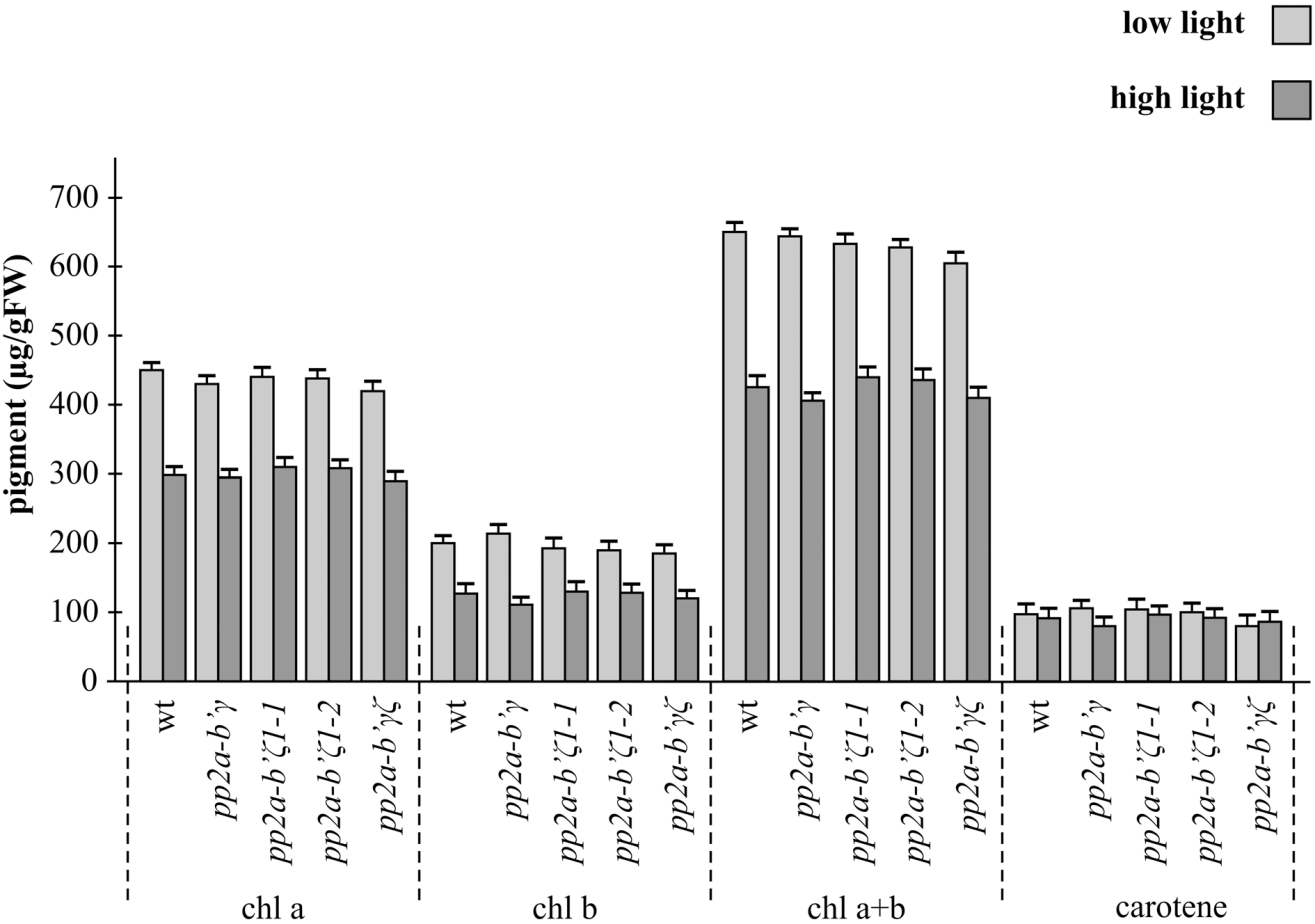
**A comparison of the leaf pigment contents in the *pp2a-b'γ, pp2a-b'ζ 1-1, pp2a-b'ζ 1-2*, and the pp2a-*b'*γζ double mutant grown under either low or high light conditions**. Chlorophyll a (chl a), chlorophyll b (chl b), total chlorophyll (chl a+b) and total carotenoid pigments (carotene) analysis was performed on the whole rosettes of plants that had been grown for 2 weeks under low light (250 μmol m^−2^ s^−1^) and then either maintained for a further 7 days under low light growth conditions or transferred to high light (800 μmol m^−2^ s^−1^) for a further 7 days.

The chlorophyll and carotenoid contents of the rosette leaves were similar in all genotypes under low light conditions (Figure 4). Growth under high light conditions for 7 days decreased leaf chlorophyll by about 30% in all genotypes relative to the leaves grown under low light conditions (Figure 4). The light-dependent decreases in leaf chlorophyll were similar in all genotypes (Figure 4).

### The composition of PP2A subunits had no effect on photosynthesis

**Figure 5:**
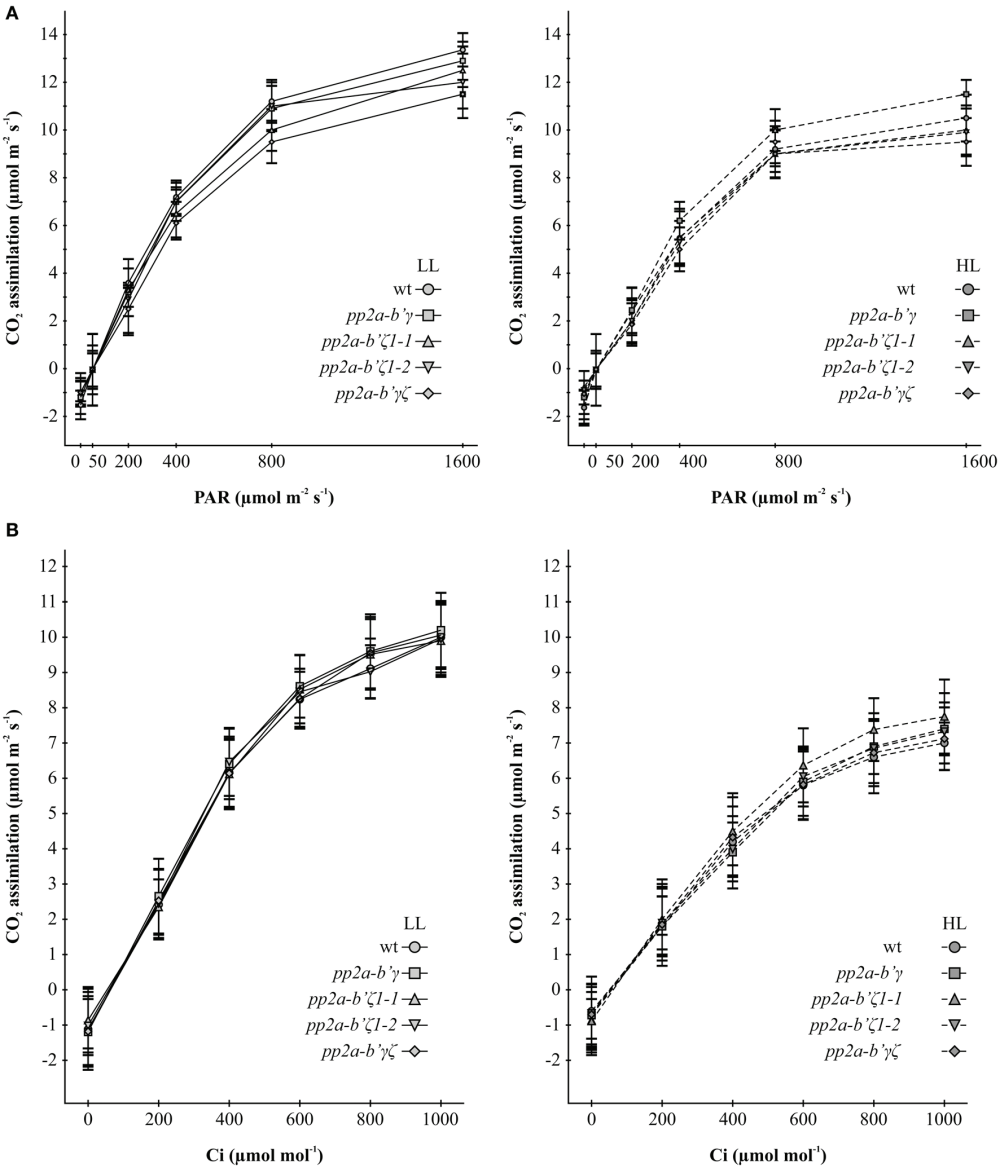
**A comparison of the light saturation curves for photosynthesis (A) and the CO_2_ response curves for photosynthesis (B) in the *pp2a-b'γ, pp2a-b'ζ1-1, pp2a-b' ζ1-2*, and the pp2a-*b*'γζ double mutant grown under either low light (LL) or high light (HL) conditions**. The light saturation curves for photosynthesis **(A)** and the CO_2_ response curves for photosynthesis **(B)** were measured on rosette leaves of plants that had been grown for 2 weeks under low light (250 μmol m^−2^ s^−1^) and then either maintained for a further 7 days under low light growth conditions or transferred to high light (800 μmol m^−2^ s^−1^) for a further 7 days.

Photosynthetic CO_2_ assimilation rates were similar in the leaves of all genotypes under low light growth conditions. Moreover, the ratio of dark-adapted variable chlorophyll a fluorescence (Fv) to maximal chlorophyll a fluorescence (Fm) was similar in all genotypes (Supplemental Figure 4). Growth under high light for 7 days decreased maximal rates of photosynthesis by about 40% relative to the leaves of plants that had been grown under low light and the Fv/Fm ratios were decreased by about 20% (Supplemental Figure 4). An analysis of the light response curves (Figure 5A) and the CO_2_ response curves for photosynthesis (Figure 5B) showed that the initial slopes of both curves were decreased in the leaves of all genotypes that had been grown under high light for 7 days compared to those that had been maintained under low light growth conditions. Moreover, there was a significant highlight dependent decrease in the CO_2_ saturated rates of photosynthesis measured in the CO_2_ response curve analysis in all genotypes (Figure 5B). The highlight dependent decrease in the light-saturated rates of photosynthesis was less marked in light response curve analysis (Figure 5A). No significant differences in these parameters were observed between the wild type and mutant lines.

### Pre-exposure to high light and the composition of PP2A subunits effects on aphid fecundity

**Figure 6:**
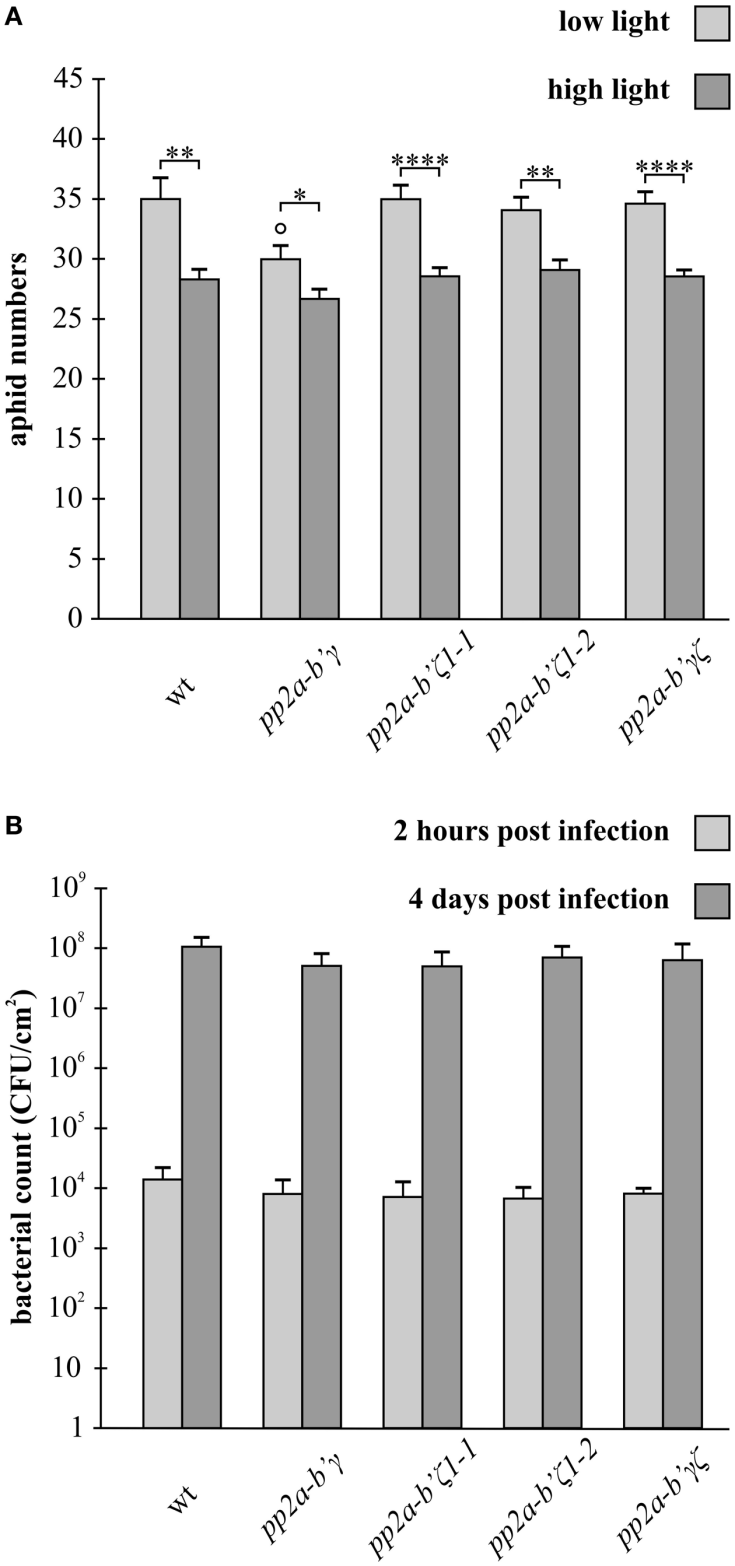
**A comparison of aphid fecundity in the *pp2a-b*'γ, *pp2a-b*'ζ 1-1, *pp2a-b'ζ 1-2*, and the pp2a-*b*'γζ double mutant plants grown under either low light or following a 7-day pre-treatment with high light (A), and the effects of these genotypes on infection by the hemibiotrophic pathogen *Pseudomonas syringae* (B)**. In **(A)**, the numbers of aphids present per plant were measured 2 weeks after the onset of infestation in plants that had either been grown for 5 weeks under low light (250 μmol m^−2^ s^−1^) or on plants that had been grown for 2 weeks under low light followed by 7 days under high light (800 μmolm^−2^ s^−1^) and then returned to the low light growth conditions prior to infestation and then grown with aphid infestation for a subsequent period of 2 weeks. The results are average values ± standard error values, *n* = 14. ^*^p < 0.05; ^**^p < 0.01; ^****^p < 0.0001 in Student's *t*-test and One-Way ANOVA comparisons between LL or HL light conditions. ° *p* < 0.05 in Student's *t*-test and One-Way ANOVA comparisons between the mutant lines and wild type in the LL or HL light conditions. In **(B)**, bacterial suspensions containing10^5^ colony forming units ml^−1^ were carefully infiltrated into two fully expanded leaves of each plant and the abundance of the pathogen (CFU) was determined on low light-grown leaves 2 h and 4 days post inoculation. The results are averages ± standard error, *n* = 5.

Aphid fecundity measured in plants that had been grown only under low light growth conditions was similar in all genotypes, except for the mutants that lack the *gamma* (*pp2a-b'γ*) subunit of PP2A phosphatase (Figure 6A). The number of aphids was significantly lower (15%) on the leaves of the *pp2a-b'γ* mutant compared to the wild type, *zeta1* (*pp2a-b'ζ 1-1*), *zeta2* (*pp2a-b'ζ 1-2*), and *gamma zeta* double mutant (*pp2a-b'γζ*). Interestingly, the decrease in aphid fecundity observed in the *gamma* (*pp2a-b'γ*) mutant was not observed in the *gamma-zeta* (*pp2a-b'γζ*) double mutant (Figure 6A).

Growth under high light conditions for 7 days prior to the analysis of aphid fecundity led to a significant light-dependent decrease (up to 20%) in aphid numbers on all genotypes (Figure 6A). While aphid fecundity was similar in all genotypes that had been exposed to the high light pre-treatments, the light-dependent decrease in aphid fecundity was least marked (11%) in the *gamma* (*pp2a-b'γ*) mutant (Figure 6A).

### The gamma and zeta subunits of PP2A have no effect on susceptibility to *pseudomonas syringae*

To evaluate whether the composition of PP2A subunits alters resistance to the hemibiotrophic bacterium, *Pseudomonas syringae* pv tomato DC3000 (Pst), we challenged leaf tissues of plants grown under an irradiance of 130 μmol m^−2^ s^−1^, to this virulent pathogen, which proliferates in the intercellular spaces of leaf tissues of wild type plants causing disease with spreading, chlorotic lesions (Pavet et al., 2005). In a previous study, infection of plants with 10^8^ colony forming units revealed no differences in bacterial growth at 2 days post-infection, and only a statistically insignificant eight-fold reduction in bacterial growth was observed at 5 days post-infection in *pp2a-b'γ* mutant compared to wild type (Trotta et al., 2011). Here we assessed bacterial growth by carefully infiltrating *Pseudomonas* in 10^5^ CFU into the leaves and quantified bacterial growth *in planta* and hence the development of pathogen, 2 h after Pst inoculation, and 4 days after Pst inoculation, a point where typical disease symptoms were observed on the leaves. Pst proliferation was similar in all genotypes at both time points (Figure 6B).

## Discussion

Plants have co-evolved with an enormous variety of fungal pathogens and insect herbivores under conditions with very different types of abiotic stresses. They therefore harbor a large reservoir of natural adaptive mechanisms to maximize growth and survival while coping with different forms of stress simultaneously. In natural environments plants experience constantly changing light levels over each day and across the seasons. Exposures to high light can be stressful to plants, triggering the innate immune responses associated with pathogen-associated molecular patterns that enhance defenses against pathogen attack (Szechynska-Hebda et al., 2010; Karpinski et al., 2012). High light can therefore elicit cross-tolerance responses to different stresses. The data presented here show that a pre-exposure to high light induces adaptations in photosynthesis and changes in plant growth that may also give rise to a molecular memory of stress, leading to an enhanced resistance to aphid infestation (but not to pathogen attack) in all the genotypes analyzed. The high light-dependent increase in growth in the mutants lacking the B'γ (*gamma*; *pp2a-b'γ*) or B'ζ (*zeta*; *pp2a-b'ζ 1-1* and *pp2a-b'ζ 1-2*) subunits or lacking both subunits is striking, and suggests that the restriction on growth imposed by the loss of the regulation of PP2A-dependent processes is overcome by highlight dependent pathways.

PP2A-B'γ regulates organellar ROS signaling and plays a key role in the negative control of SA-linked responses and associated metabolic alterations in *A. thaliana* (Trotta et al., 2011; Li et al., 2014). Metabolite profiling analysis has shown that the *pp2a-b'γ* mutation has only a minor impact on the contents of amino acids and sugars in non-stressed plants, with only slight increases in the levels of homoserine and tryptophan and reduced levels of malic acid and citric acid in *pp2a-b'*γ leaves (Li et al., 2014). However, when combined with a mutation in the photorespiratory form of *catalase 2* (*cat2*) in a double mutant, the *pp2a-b'γ* mutation increases the *cat2*-triggered accumulation of amino acids and camalexin, suggesting that PP2A-B'*γ* may influence pathways leading to secondary metabolism in response to oxidative signals (Li et al., 2014). This finding is consistent with observations demonstrating that PP2A-B'*γ* physically interacts with the cytoplasmic form of ACONITASE 3, a metabolic enzyme that is functionally associated with respiration, oxidative stress responses and cell death (Konert et al., authors own unpublished data). Further analysis of previously published microarray data revealed that SA signaling and cell death pathways are increased in *pp2a-b'γ* relative to the wild type (Trotta et al., 2011). Moreover, while no transcripts related to callose synthesis were differentially increased in the *gamma* (*pp2a-b'γ*) mutant (Trotta et al., 2011), the abundance of mRNAs encoding the beta-glucanase pathogenesis-related (PR) protein 2, which negatively regulates the deposition of the callose, was increased relative to the wild type, together with other PR transcripts.

The data presented here provides the first evidence that PP2A-B' negatively controls plant resistance to aphids in low light-grown *A. thaliana* plants, and that this regulatory pathway is functionally connected with PP2A-B'ζ, which in turn seems to have a positive impact on defense signaling (Figure 6A). While knock-down *pp2a-b'γ* mutants show decreased aphid fecundity under low light growth conditions, the *pp2a-b'γζ* double mutant supports aphid propagation in a similar manner to that observed in the wild type plants (Figure 6A). It should be noted that to date these observations have been made only on one mutant line because there are no other suitable mutant alleles available for analysis. Similarly, there are no transformed plants with appropriate RNAi constructs available as yet for these subunits. However, it is well known that PP2A phosphatases with different subunit compositions may regulate signaling networks at multiple nodes in both animals and plants (Hardie, 1990; Tang et al., 2011; Wu et al., 2011). Such multi-level action is possible because PP2A may assemble in a large number of different heterotrimeric holoenzymes with different functional properties and therefore high specificity toward specific target phospho-proteins.

The computational models of heterotrimeric PP2A complexes described here suggest that PP2A-B'γ and PP2A-B'ζ may bind similar PP2A-A/C dimers (Figure 1C). Hence, when PP2A-B'γ is absent, PP2A-B'ζ can act as a positive mediator and may take over to promote defensive processes. Indeed, the perturbations in both regulatory subunits that occurs in the *pp2a-b'γζ* double mutant, appear to revert the situation back to the wild type (Figure 6A). Since the PP2A A and C subunits share high amino acid sequence identity (Supplemental Figures 1, 2), the variable regulatory B subunits mediate essential roles in determining the substrate specificity and subcellular targeting of PP2A (Matre et al., 2009; Uhrig et al., 2013). Fluorescence-tagged versions of both B'γ and B'ζ have been observed in the cytoplasm. Moreover, B'ζ was localized to mitochondria (Matre et al., 2009; Trotta et al., 2011). Distinct functions of the GAMMA and ZETA subunits could also be mediated by the variable N-terminal domains that extrude from the core of the PP2A holoenzyme. The N-terminal domain may therefore determine interactions with other proteins, which because of the unique characteristics of the amino acid sequences are likely to differ between PP2A-B'γ and PP2A-B'ζ. Trimeric PP2A holoenzymes with B'γ or B'ζ may therefore regulate cellular functions in seemingly opposite ways. Furthermore, it is probable that both the GAMMA and ZETA subunits interact with a number of target phospho-proteins and therefore serve multiple functions, which might be changed in the mutants analyzed here. It is also possible that other B' subunits could replace the B' subunits in the *gamma zeta* double knockout because a number of different B' subunits will be available. However, higher order mutant combinations would be required to investigate this point.

### Conflict of interest statement

The authors declare that the research was conducted in the absence of any commercial or financial relationships that could be construed as a potential conflict of interest.

## Supplementary material

Supplemental Figure 1 **Amino acid sequence alignments of PP2A-B subunits**. Seventeen known Arabidopsis PP2A-B subunits are aligned with human PP2A-B56γ.

Supplemental Figure 2 **Amino acid sequence alignment of Arabidopsis PP2A-A subunits**. The mouse α subunit from the X-Ray structure of the known PP2A trimer (PDB code: 3FGA, chain A) is shown in parallel. Sequence similarity, based on the three Arabidopsis sequences, is highlighted.

Supplemental Figure 3 **Amino acid sequence alignment of Arabidopsis PP2A-C subunits**. The human catalytic subunit from the X-Ray structure of the known PP2A trimer (PDB code: 3FGA, chain C) is shown in parallel. Sequence similarity is highlighted.

Supplemental Figure 4 **The change in the ratio of dark-adapted variable chlorophyll a fluorescence (Fv) to maximal chlorophyll a fluorescence (Fm) observed following the transfer of plants grown under low light to high light for 7 days**.
