# Supplementary figures and images for: Effects of light and the regulatory B-subunit composition of protein phosphatase 2A on the susceptibility of Arabidopsis thaliana to aphid (Myzus persicae) infestation
Source: Front Plant Sci. 2014 Aug 21;5:405. doi: 10.3389/fpls.2014.00405 (PMC4140078; doi:10.3389/fpls.2014.00405)

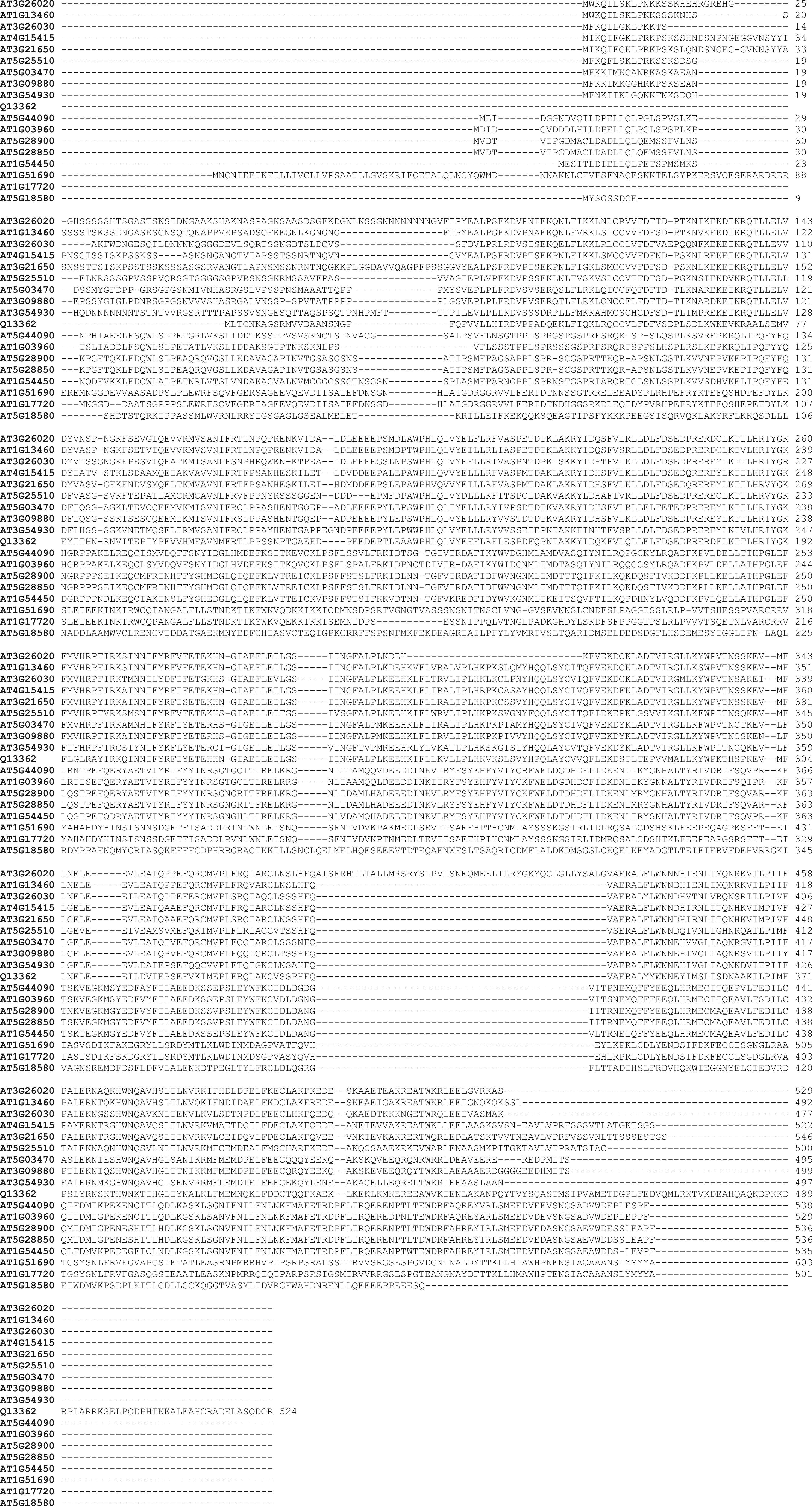

Supplement: Supplemental Figure 1 — Amino acid sequence alignments of PP2A-B subunits. Seventeen known Arabidopsis PP2A-B subunits are aligned with human PP2A-B56γ. [file Image1.JPEG]

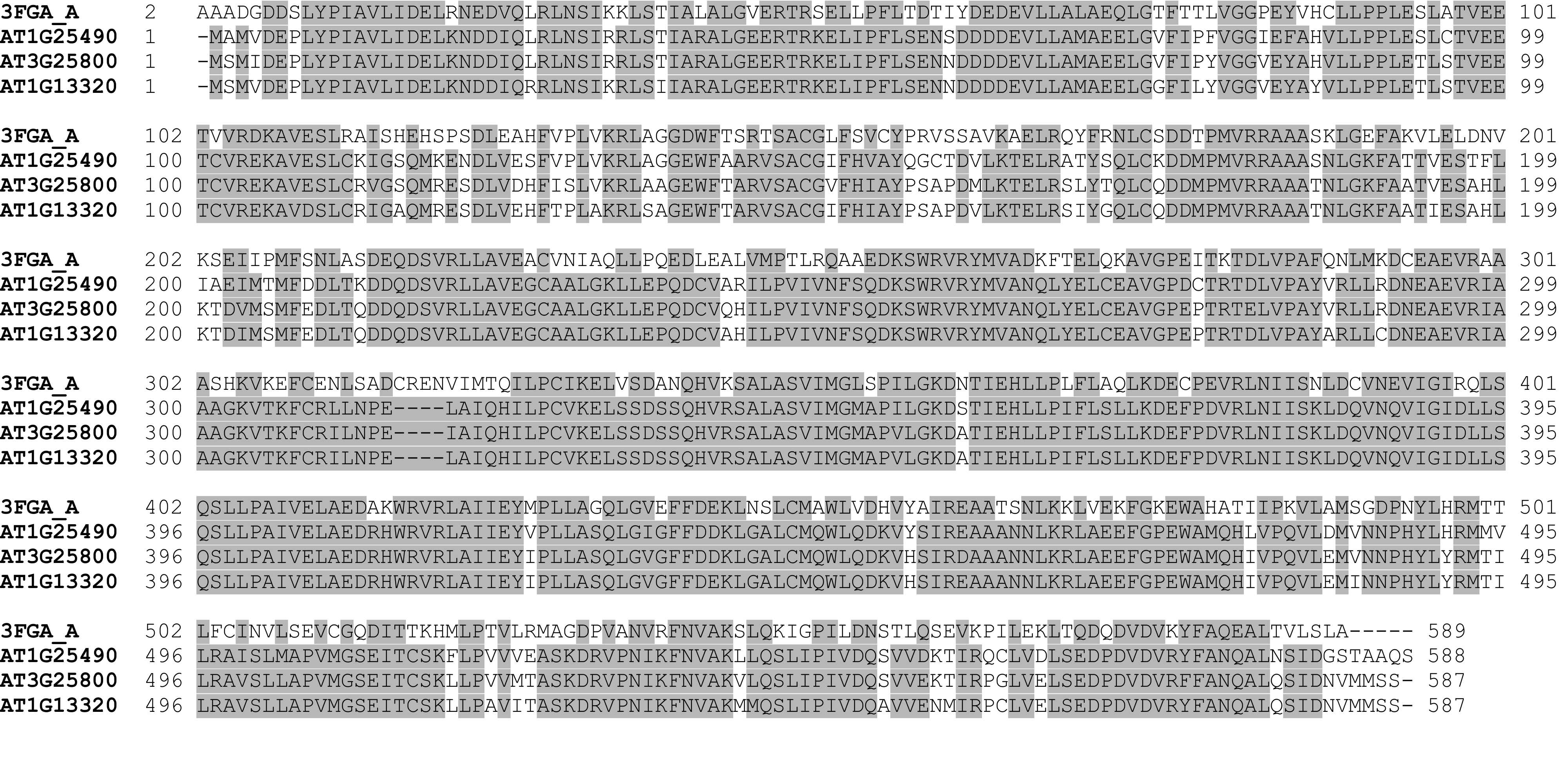

Supplement: Supplemental Figure 2 — Amino acid sequence alignment of Arabidopsis PP2A-A subunits. The mouse α subunit from the X-Ray structure of the known PP2A trimer (PDB code: 3FGA, chain A) is shown in parallel. Sequence similarity, based on the three Arabidopsis sequences, is highlighted. [file Image2.JPEG]

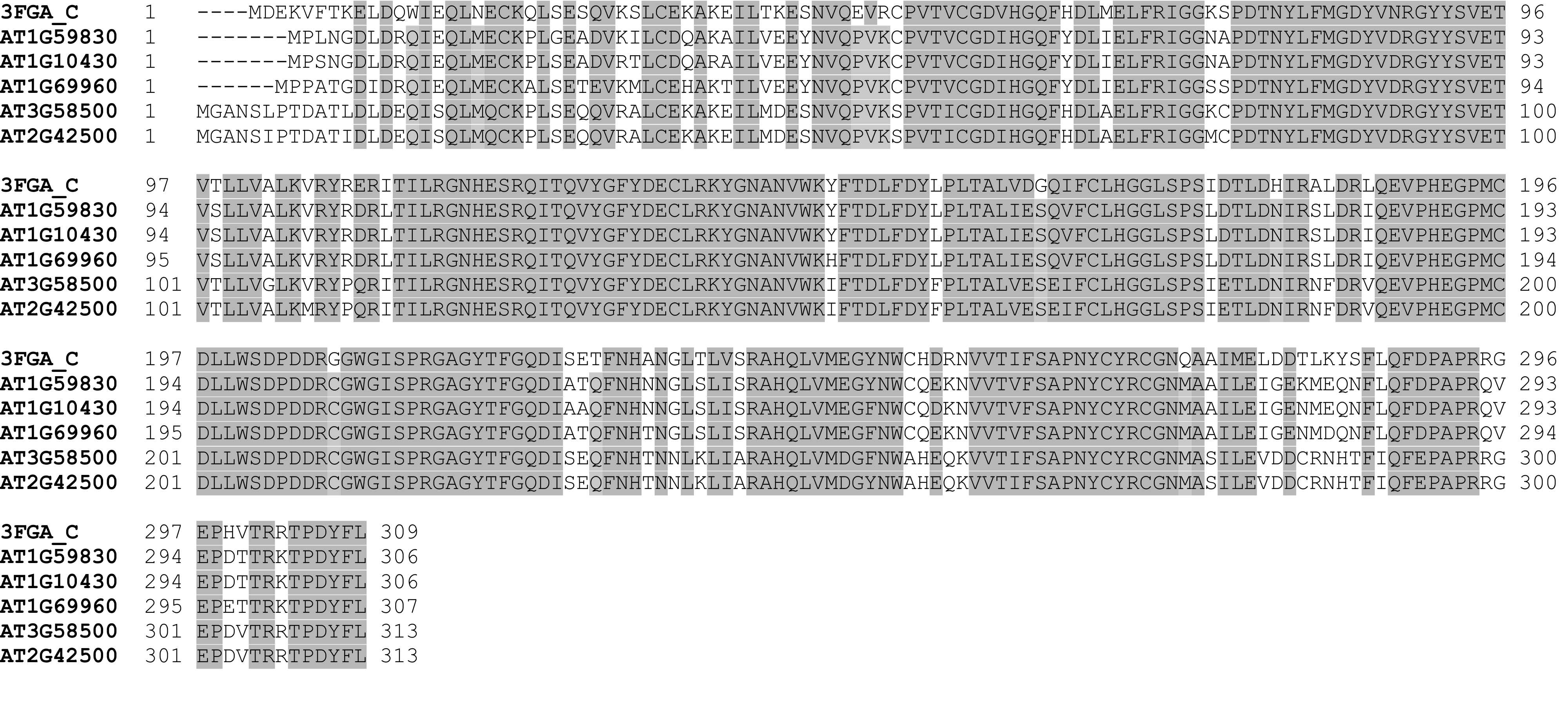

Supplement: Supplemental Figure 3 — Amino acid sequence alignment of Arabidopsis PP2A-C subunits. The human catalytic subunit from the X-Ray structure of the known PP2A trimer (PDB code: 3FGA, chain C) is shown in parallel. Sequence similarity is highlighted. [file Image3.JPEG]

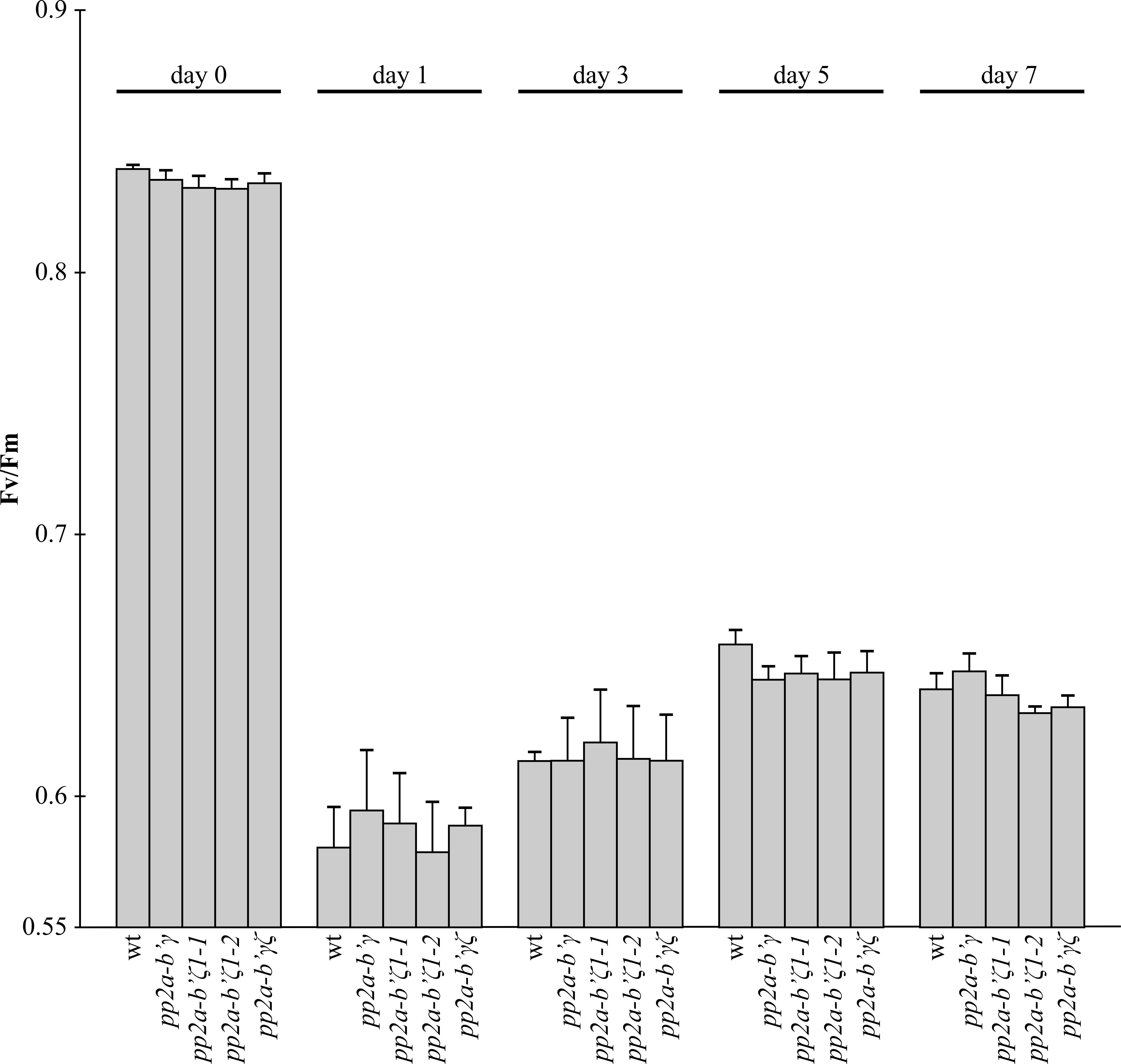

Supplement: Supplemental Figure 4 — The change in the ratio of dark-adapted variable chlorophyll a fluorescence (Fv) to maximal chlorophyll a fluorescence (Fm) observed following the transfer of plants grown under low light to high light for 7 days. [file Image4.JPEG]
